# Brain Site-Specific Inhibitory Effects of the GLP-1 Analogue Exendin-4 on Alcohol Intake and Operant Responding for Palatable Food

**DOI:** 10.3390/ijms21249710

**Published:** 2020-12-19

**Authors:** Kayla J. Colvin, Henry S. Killen, Maxwell J. Kanter, Maximilian C. Halperin, Liv Engel, Paul J. Currie

**Affiliations:** Department of Psychology, Reed College, 3203 SE Woodstock Blvd., Portland, OR 97202, USA; colvink@reed.edu (K.J.C.); henryskillen@gmail.com (H.S.K.); kanterm@reed.edu (M.J.K.); maxhalpe@reed.edu (M.C.H.); engelol@alumni.reed.edu (L.E.)

**Keywords:** appetitive behavior, ethanol, exendin-4, glucagon-like peptide 1, mesocorticolimbic, mesolimbic, reward

## Abstract

Approximately 14.4 million Americans are experiencing alcohol use disorder (AUD) and about two-thirds of people who experience drug addiction will relapse, highlighting the need to develop novel and effective treatments. Glucagon-like peptide-1 (GLP-1) is a peptide hormone implicated in the mesocorticolimbic reward system and has become a peptide of interest with respect to its putative inhibitory effects on drug reward. In order to further develop treatments for those diagnosed with AUD, the interplay between GLP-1 receptor signaling and ethanol consumption must be elucidated. In the present study, we investigated the ability of the GLP-1 analogue, exendin-4 (Ex-4), to alter alcohol intake and operant responding for sucrose pellets in order to further understand the role of this compound in mediating reward. We selected multiple sites throughout the prosencephalic and mesencephalic regions of the brain, where we directly administered various doses of Ex-4 to male Sprague Dawley rats. In alcohol investigations, we utilized a two-bottle choice intermittent access protocol. In separate groups of rats, we adopted an operant paradigm in order to examine the effect of Ex-4 on motivated responding for palatable food. Results indicated that GLP-1 receptor signaling effectively suppressed voluntary alcohol intake when injected into the ventral tegmental area (VTA), the accumbens core (NAcC) and shell (NAcS), the dorsomedial hippocampus (DMHipp), and the lateral hypothalamus (LH), which are all structures linked to brain reward mechanisms. The arcuate nucleus (ARcN) and the paraventricular nucleus (PVN) of the hypothalamus were unresponsive, as was the basolateral amygdala (BLA). However, Ex-4 treatment into the ArcN and PVN suppressed operant responding for sucrose pellets. In fact, the VTA, NAcC, NAcS, LH, and the DMHipp all showed comparable suppression of sucrose responding. Overall, our findings suggest that these central structures are implicated in brain reward circuitry, including alcohol and appetitive motivation, which may be mediated by GLP-1 receptor mechanisms. GLP-1, therefore, may play a critical role in modifying addictive behaviors via activation of multiple GLP-1 systems throughout the brain.

## 1. Introduction

Alcohol is the most widely used and abused intoxicant in the United States, with 139.8 million Americans aged 12 and older having consumed alcohol in the past month and an estimated 14.4 million Americans in the United States aged 12 and older (5.4% of the U.S. population) experiencing alcohol use disorder (AUD) in 2018 [1]. AUD is characterized by an impaired ability to limit or control alcohol use despite adverse health, occupational, and social consequences [2]. Despite advances in pharmacological, psychological, and social interventions for AUD, more than 60% of individuals diagnosed with AUD revert back to hazardous alcohol use post-treatment [3]. Given the limited success of current approved medications for AUD, it is prudent for novel pharmaceutical treatments to be developed [4,5]. In recent years, gut-brain peptides, such as glucagon-like peptide-1 (GLP-1), typically involved in appetitive functions, have been implicated in the mesolimbic reward system and likewise proposed as novel treatments for AUD [6,7]. GLP-1 is a 30-amino acid peptide hormone derived from preproglucagon and produced in the L-type enteroendocrine cells in response to food intake [8]. GLP-1 binds to and activates GLP-1 receptors (GLP-1R), which are a B family of G-protein-coupled receptors (GPCR) [9]. GLP-1 serves numerous metabolic functions, including mediating glucose-dependent stimulation of insulin secretion, decreasing gastric emptying, and inhibiting food intake, making GLP-1 agonists novel treatments for Type-2 and Type-3 diabetes [10,11,12].

In the central nervous system (CNS), pre-glucagon expression is found in the nucleus of the solitary tract (NTS), where GLP-1 is secreted via neuronal projections throughout the CNS, namely areas involved in homeostatic and hedonic eating behaviors [13,14]. GLP-1 receptors are highly expressed in hypothalamic nuclei in rodents and non-human primates, including the arcuate nucleus of the hypothalamus (ArcN), the paraventricular nucleus (PVN), and the lateral hypothalamus (LH) [13,14,15,16,17]. Central activation of GLP-1 receptors in the LH and PVN has been found to significantly reduce food intake in rats [18,19]. Furthermore, GLP-1R signaling in the ArcN and PVN via treatment with the GLP-1 receptor agonist, exendin-4 (Ex-4), decreases metabolism and attenuates ghrelin-stimulated increases in metabolic activity [20,21]. Together, these findings suggest that GLP-1 analogues have an anorexigenic effect when administered into the hypothalamus.

GLP-1 receptors are also expressed in areas of the mesocorticolimbic reward circuit, including the amygdala, hippocampus, ventral tegmental area (VTA), and nucleus accumbens (NAc) [13,14,15,16,17]. The central amygdala (CeA) and basolateral amygdala (BLA) have been broadly implicated in food-related behaviors [22,23]. Prior studies found that lesions of the CeA attenuate Ex-4-induced decreases in consumption of palatable food and that the anorexigenic effects of GLP-1 receptor signaling are partially mediated by intra-amygdala dopamine transmission [24,25]. Within the hippocampus, GLP-1R activity modulates CA1 hippocampal activity in rats by increasing rates of neuronal firing [26]. When injected into the ventral hippocampal formation (HPFv), Ex-4 attenuates food intake and various food-motivated behaviors [27,28]. GLP-1 receptor signaling also mediates hedonic eating behavior at the level of the VTA and NAc [29,30,31,32,33]. Central injection of Ex-4 into the VTA and NAc has been found to reduce food intake [29] and attenuate the establishment of reward-related memory measured by palatable food-induced conditioned place preference (CPP) [30]. This effect is further supported by the increase in operant responding for palatable food upon administration of the GLP-1 receptor antagonist, exendin-9 (Ex-9), into the NAc core (NAcC) [32]. Based on the research outlined above, it is believed that GLP-1 signaling within areas of the mesocorticolimbic pathway mediates reward-related appetitive behaviors.

Consumption of alcohol and other rewarding substances may be regulated by mechanisms similar to those that regulate hedonic eating behaviors. Peripheral administration of different GLP-1 analogues in rodents and non-human primates suppresses various alcohol-mediated responses, including operant consumption of alcohol [34,35,36,37,38], CPP [34,35,37], alcohol intake [34,37,39], alcohol deprivation, and accumbal dopamine release [34,37]. These findings substantiate the relevancy of GLP-1 agonists as potential and viable treatments for AUD, but more research is necessary to better understand the exact mechanisms that mediate the relationship between GLP-1 receptor activation and alcohol reward. Studies examining the effect of the central administration of GLP-1 agonists have begun to map out this circuit. For example, intra-NTS Ex-4 inhibits alcohol-induced accumbal dopamine release, locomotor stimulation, and CPP [40]. Other findings suggest that GLP-1 receptor signaling within the VTA significantly reduces alcohol self-administration but not alcohol reacquisition [35,41]. Interestingly, another study found that a bilateral central injection of Ex-4 into the anterior VTA (aVTA) had no effect on alcohol-induced behaviors and only inhibited locomotor stimulation, but not alcohol intake or CPP, when injected into the posterior VTA (pVTA) [42]. Research in our lab has also demonstrated that unilateral central administration of Ex-4 targeting the NAc shell (NAcS) reduced alcohol intake in female rats [43]. Vallöf et al. [42] reported similar results, where injection Ex-4 at the level of the NAcS blocked alcohol-induced locomotor stimulation and CPP while reducing overall alcohol intake.

The purpose of the present study was to investigate the impact of Ex-4, a GLP-1 analogue, on the rewarding properties of palatable food and alcohol consumption when administered into multiple brain areas involved in mesocorticolimbic reward circuitry and homeostatic mechanisms associated with motivation. We sought to replicate and extend previous research regarding GLP-1 analogues and their impact on the mesocorticolimbic reward circuit. We investigated the effects of GLP-1 receptor activation on alcohol intake via two-bottle choice and operant responding for palatable food through central injections of Ex-4 into sites directly and indirectly associated with brain reward and appetitive circuitry. These structures included the ventral tegmental area (VTA), the nucleus accumbens core (NAcC), the nucleus accumbens shell (NAcS), the basolateral region of the amygdala (BLA), and the dorsomedial hippocampus (DMHipp), as well as the hypothalamic arcuate nucleus (ArcN), the paraventricular nucleus (PVN), and the lateral hypothalamus (LH). Taken together, our results present compelling evidence for GLP-1’s integral role in mediating appetitive motivation and drug reward.

## 2. Results

### 2.1. Alcohol Consummatory Studies

**Figure 1:**
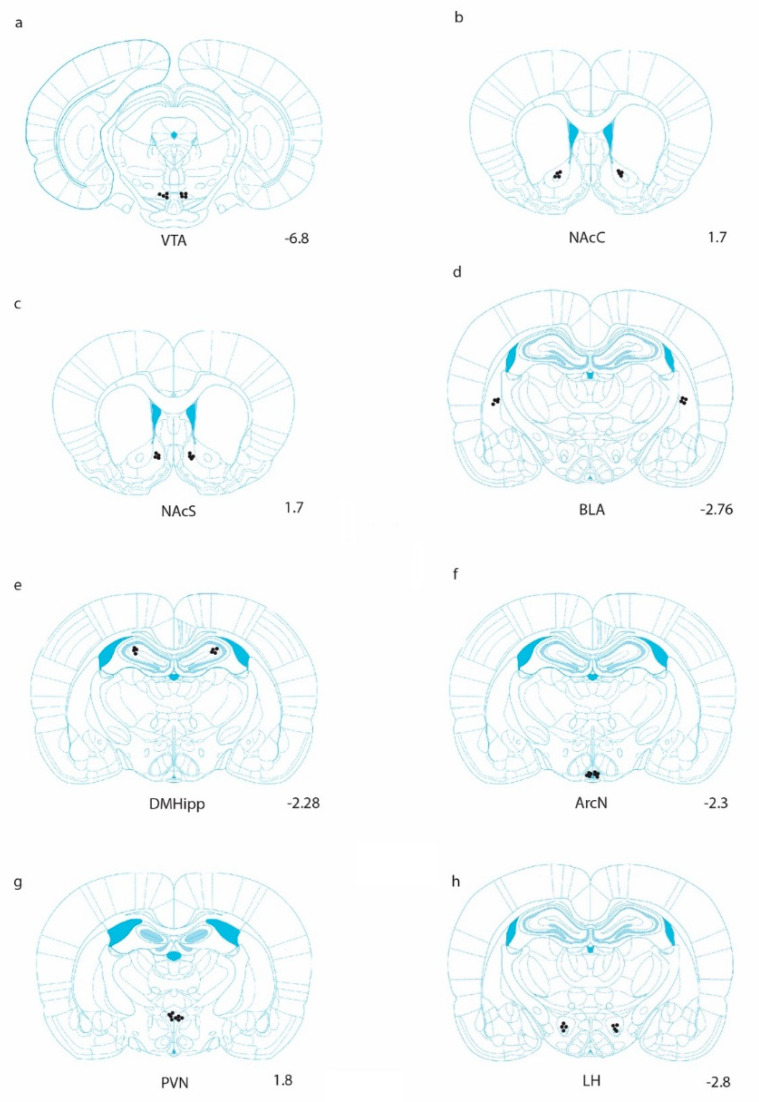
Anatomical representation of injection sites in which Ex-4 was unilaterally delivered. Injections were counterbalanced in the left and right hemispheres [44]. Coronal sections are shown for (**a**) the ventral tegmental area (VTA), (**b**) the nucleus accumbens core (NAcC), (**c**) the nucleus accumbens shell (NAcS), (**d**) the basolateral amygdala (BLA), (**e**) the dorsomedial hippocampus (DMHipp), (**f**) the arcuate nucleus (ArcN), (**g**) the paraventricular nucleus (PVN), and (**h**) the lateral hypothalamus (LH).

**Figure 2:**
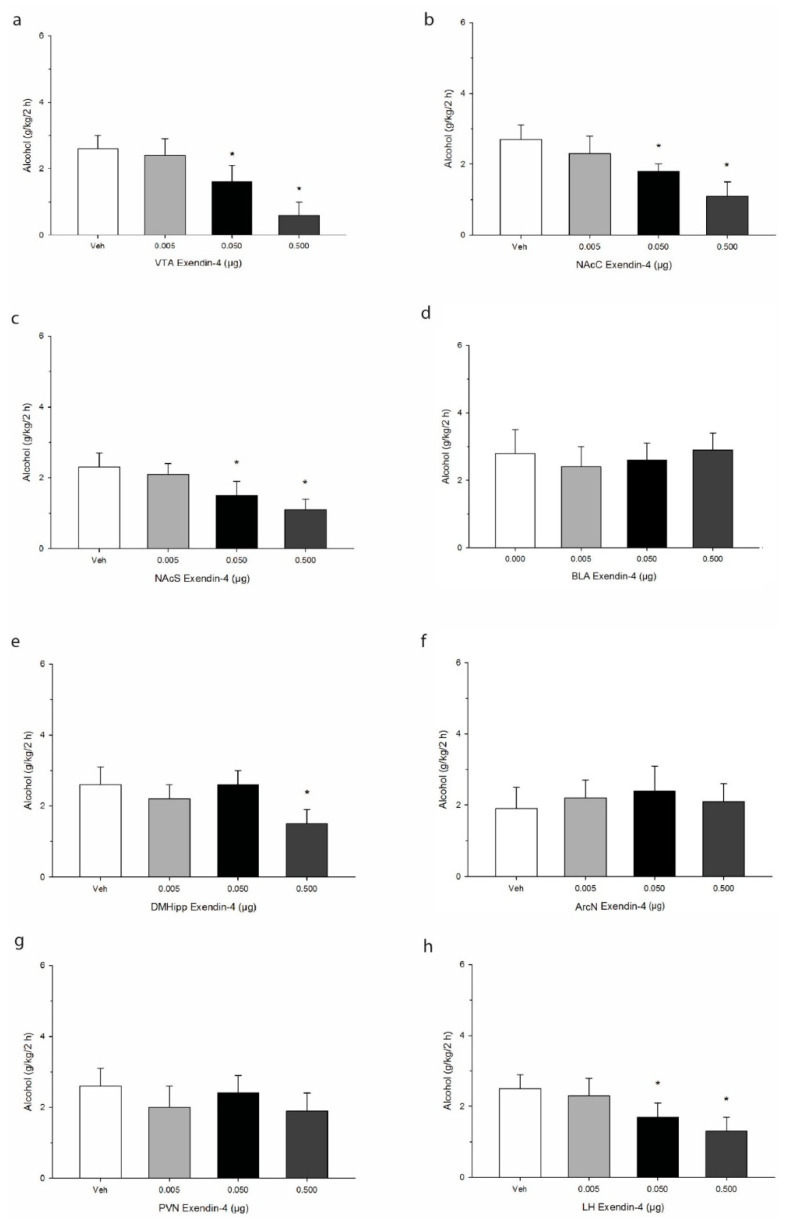
Effects of microinjection of Ex-4 on alcohol intake over a 2 h period in the early nocturnal cycle. Treatment was delivered into primary regions of the mesolimbic system, including (**a**) the ventral tegmental area (VTA), (**b**) the nucleus accumbens core (NAcC), and (**c**) the nucleus accumbens shell (NAcS). Injections were also administered into (**d**) the basolateral amygdala, (**e**) the dorsomedial hippocampus (DMHipp), and discrete regions of the hypothalamus, specifically (**f**) the arcuate nucleus (ArcN), (**g**) the paraventricular nucleus (PVN), and (**h**) the lateral hypothalamus (LH). Values represent mean alcohol intake +/− SEM. * *p* < 0.05 compared to Veh; *n* = 8/site.

Cannula placement was confirmed using histological verification as we have previously described [43]. A schematic representation of targeted sites is shown in Figure 1. Data were analyzed by separate one-way analysis of variances (ANOVA) followed by post hoc Tukey where justified. When injected into the VTA, Ex-4 elicited reliable decreases in 2 h alcohol consumption (g/kg/2 h), and post hoc analysis indicated that the effect was significant at the two higher doses administered F(3,21) = 50.7, *p* < 0.0001. See Figure 2. Similar robust effects were found upon injection into both the core and shell regions of the accumbens (NAcC, F(3,21) = 37.7, *p* < 0.001; NAcS F(3,21) = 34.6, *p* < 0.001). In the BLA, Ex-4 had no reliable impact on alcohol intake at any of the doses tested F(3,21) = 9.1, *p* > 0.05. In the DMHipp, the highest dose of 0.5 µg evoked a significant decrease in intake F(3,21) = 22.1, *p* < 0.01. In contrast no effect of Ex-4 was observed on alcohol intake after injection into either the ArcN F(3,21) = 3.1, *p* > 0.05 or PVN F(3,21) = 4.6, *p* > 0.05. Finally, in the LH, Ex-4 robustly suppressed alcohol intake F(3,21) = 39.3, *p* < 0.001, suggesting GLP-1 mediates alcohol consumption within this region.

### 2.2. Operant Conditioning Paradigms

**Figure 3:**
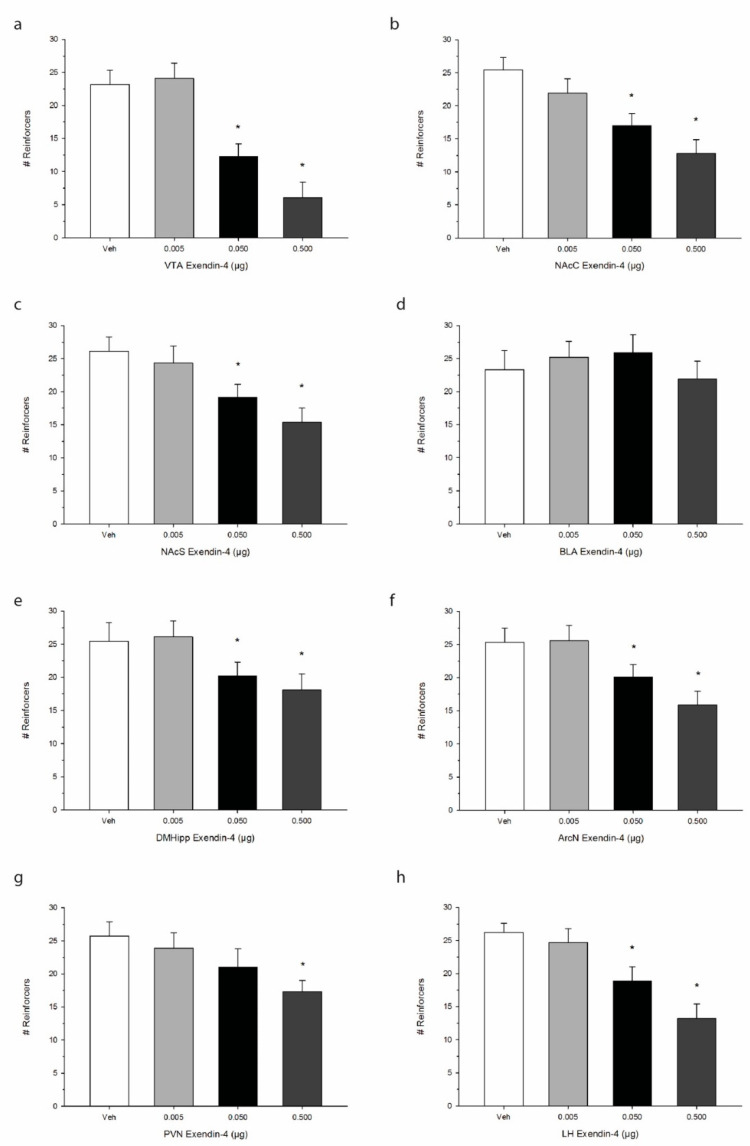
Effects of direct injections of Ex-4 on operant responding for palatable sucrose pellets. Values refer to the mean number of reinforcers obtained +/− SEM. We observed site-specific alterations in the inhibitory effect of Ex-4. Brain regions including (**a**) the ventral tegmental area (VTA), (**b**) the nucleus accumbens core (NAcC), and (**c**) the nucleus accumbens shell (NAcS) as well as (**e**) the dorsomedial hippocampus (DMHipp), (**f**) the arcuate nucleus (ArcN), (**g**) the paraventricular nucleus (PVN), and (**h**) the lateral hypothalamus (LH), exhibited an anorexigenic effect of Ex-4 on sucrose consumption. Operant responding in (**d**) the basolateral amygdala (BLA), however, was not impacted. * *p* < 0.05 compared to Veh; *n* = 8/site.

The effects of Ex-4 treatment on operant responding for palatable food are shown in Figure 3. One-way repeated measures ANOVA indicated that Ex-4 significantly suppressed the total number of reinforcers when injected into the VTA, representing a decrease in operant responding for food reward or palatability F(3,21) = 62.8, *p* < 0.0001. We observed similar robust effects of Ex-4 in suppressing total reinforcers when injected into the NAcC F(3,21) = 41.6, *p* < 0.001 as well as the NAcS F(3,21) = 52.7, *p* < 0.0001. While Ex-4 treatment did not alter operant responding when delivered into the BLA F(3,21) = 3.4, *p* > 0.05, DMHipp treatment was found to suppress intake at the two higher doses of Ex-4 F(3,21) = 38.9, *p* < 0.001. Additionally, Ex-4 administration into all three hypothalamic sites elicited reductions in operant responding. Specifically, ArcN Ex-4 treatment decreased total reinforcers F(3,21) = 40.2, *p* < 0.001, as did PVN F(3,21) = 36.8, *p* < 0.001, and LH treatment F(3,21) = 50.7, *p* < 0.0001. Interestingly, while Ex-4 suppressed operant responding for food in all three hypothalamic sites, ArcN and PVN Ex-4 administration had no observable effects on alcohol consumption as noted above.

## 3. Discussion

The goal of the current research was to map GLP-1 activity throughout the central nervous system in the context of motivated food-seeking and alcohol consumption. Previous research has revealed that GLP-1 plays a critical role in learning, metabolism, motivation, and reward; our goal was to map where these interactions take place in the CNS. Our data support previous findings that GLP-1 analogues reliably decrease both perceived reward and motivation to receive rewards when centrally administered to the NAc and VTA [30,34,45,46,47]. More specifically, we observed a reduction in both operant responses to banana-flavored sucrose pellets and alcohol consumption when Ex-4 was administered to the VTA, NAcC, NAcS, DMHipp, and LH. Operant responding for sucrose pellets was also reduced by infusion of Ex-4 into the PVN and ArcN, with no observed effects on alcohol consumption. Lastly, we found no effect of injection into the BLA at any of the doses tested for both food-driven operant behavior and alcohol consumption. These findings suggest that GLP-1 has a profound influence on mesolimbic dopamine signaling during both reward motivated behavior and alcohol-consummatory behavior. Additionally, Ex-4 is active both in the hypothalamus, where it may stimulate food-seeking via changes in metabolism, and in the hippocampus, where it may affect learning of reward evoked cues.

Previous research strongly suggests that GLP-1 signaling is able to modulate incentive value via action along the central mesolimbic reward pathway. Jerlhag and colleagues found that when injected peripherally, Ex-4 was capable of reducing amphetamine-induced locomotor stimulation, CPP, and accumbal dopamine release [45]. Sørensen and colleagues showed that peripherally administered Ex-4 reduced cocaine-induced locomotor stimulation and cocaine intravenous self-administration (IVSA) [47]. They further demonstrated that when injected directly into either the VTA or NAc, Ex-4 attenuated both palatable food consumption and progressive ratio (PR) reached in an operant setting. We previously showed that central Ex-4 administration into the VTA attenuates ghrelin’s well-documented increase in operant responding for sucrose pellets [33,48,49,50]. Taken together, these data indicate that Ex-4 is not only involved in the regulation of dopamine release but is critically involved in reward processing and motivational salience.

In alignment with our data, GLP-1 agonists have previously been shown to regulate food reward and motivated behavior specifically. Dickson and colleagues found that when injected intraperitoneally (IP), Ex-4 significantly attenuates both the breakpoint during an operant paradigm for sucrose and CPP induced by palatable food [30]. As indicated above, we have previously demonstrated that pretreatment with Ex-4 either peripherally or directly into the VTA diminished responding for sucrose pellets [33]. Interestingly, we previously observed that a low dose of Ex-4, 0.01 µg, was not enough to induce a significant change in responding on its own, but it was able to attenuate the expected increase in responding following injection of ghrelin into the VTA [33]. Here we show that doses of 0.05 µg and 0.5 µg administered directly to the VTA, NAcC, or NAcS both triggered a robust decrease in operant responding for sucrose pellets. This suggests that the threshold dose for Ex-4 to noticeably affect motivated behavior via mesolimbic circuitry falls between 0.01 µg and 0.05 µg, although further research should be considered in order to establish the exact threshold. In contrast to our results, Schmidt et al. [51], reported that a dose of 0.05 µg Ex-4 injected into the VTA significantly decreased cocaine IVSA but had no effect on responding for sucrose pellets. It is important to note that in contrast to Schmidt and colleagues, our rats were not food-deprived and were tested during their dark cycle. This was done to take into account the circadian-rhythm-dependent bioavailability of appetitive peptides and their receptors. Thus, the differences in methodology between our studies could explain why we observed an effect of Ex-4 at the 0.05 µg dose. One could argue that conducting testing during the dark cycle is more representative of natural food- and reward-seeking behavior.

Our findings add to the body of evidence that Ex-4 is capable of reducing alcohol consumption and reward in mesolimbic structures. Jerlhag and colleagues found that systemic treatment with Ex-4 attenuated alcohol intake, alcohol-induced locomotor stimulation, alcohol-induced accumbal dopamine release, and alcohol-induced CPP [34]. Peripheral Ex-4 attenuated operant alcohol IVSA by 70% in mice that previously self-administered to the point of toxicity [36]. Prolonged treatment with Ex-4 reduced relapse-like behavior in alcohol-deprived rats [52]. A dose of 0.025 µg Ex-4 infused bilaterally into the NAcS resulted in lower alcohol consumption, CPP, and locomotor stimulation [42]. Similarly, we found that doses of 0.05 µg and 0.5 µg Ex-4 infused directly into the VTA, NAcC, or NAcS greatly reduced alcohol consumption in a two-bottle choice paradigm. These data refine our understanding of Ex-4 activity and demonstrate that the previously found reductions in various measures of reward following peripheral administration can be achieved via VTA or NAc activity alone. Thus, Ex-4 certainly is able to modify perceived alcohol reward via mesolimbic dopamine activity. In contrast to our results, Vallöf et al. [42] found that injection of Ex-4 into either the aVTA or pVTA had no effect on alcohol consumption or alcohol CPP but that injection into the pVTA was able to reduce alcohol-induced locomotor stimulation. The authors argue that previous studies used larger doses (0.1 µg from [35]) of Ex-4, which may reduce locomotor function and water intake by acting on non GLP-1 receptor mechanisms [30]. However, in our study, we used a comparable dose to Vallöf et al. [42] and observed significant results for both alcohol consumption and food-motivated behavior.

After observing that intra-VTA, intra-NAcS, and intra-NAcC Ex-4 injections were capable of producing a decline in both operant activity for palatable food and alcohol consumption via two-bottle choice paradigms, we decided to investigate other regions of the central nervous system where GLP-1 activity has previously been reported. The hippocampus, specifically the ventral hippocampal formation (HPFv), has previously been identified as a potential region where GLP-1 may impact eating behavior. GLP-1Rs have been found throughout the hippocampus but most strongly in the CA3 region [13]. One study found that 0.03 µg or 0.06 µg Ex-4 infused into the ventral hippocampus decreased food consumption without producing conditioned flavor avoidance and decreased motivated responding for food without impacting CPP [27]. They followed up on these results three years later when they selectively knocked down GLP-1R to identify an HPFv-medial prefrontal cortex pathway, which resulted in ablation of the decline in food consumption and weight loss normally associated with GLP-1 activity in this area [28]. Following these reports, we decided to investigate GLP-1 activity in the DMHipp. The DMHipp, including the CA3 region, has previously been associated with learning contextual cues during drug-seeking and relapse [53,54,55], although little research has connected GLP-1 activity in this area to reward- and drug-seeking behavior. We found that only our higher dose (0.5 µg) infused into the DMHipp (within the CA3 region) produced a significant decline in food-motivated behavior and alcohol preference. This suggests that GLP-1 activity may additionally be important for the learning of reward-predictive cues.

Previous research found that lesioning of the central amygdala ablated the effects of peripheral injection of Ex-4 on palatable food consumption [25]. Because of its relevance during reward-seeking behavior, we decided to examine the effects of direct injection into the BLA. The BLA, which projects to the NAcC, is critical for reward-seeking behavior [56,57]. Furthermore, long-term ethanol consumption alters several mRNAs in the BLA of rats [58], and previous research suggests that the BLA is necessary for stress-induced drug-seeking and drug-taking behavior [59]. However, we found no effect of high or low doses of Ex-4 on either alcohol consumption or food-motivated behavior in this area. Thus, the role of GLP-1 signaling in the amygdala during reward processing and seeking behavior remains to be established, especially given the lack of extensive evidence for GLP-1R expression in the BLA.

It is additionally well established that GLP-1 is metabolically active throughout the hypothalamus in regions such as the LH, PVN, and ArcN [13,14,15,16,17]. Previous research found that either 0.05 µg or 0.15 µg Ex-4 infused into the LH produced significant reductions in operant behavior for sucrose pellets and that GLP-1 antagonism via Ex-9 increased operant responding for food [60]. Our data replicate these findings; we show that either 0.05 µg or 0.5 µg Ex-4 injected centrally into the LH produced a significant decline in responding during a sucrose-driven operant paradigm. Further, we observed decreased operant responding for sucrose pellets when Ex-4 was administered into the ArcN or PVN [21,61].

Our findings are consistent with emerging evidence that projections from the LH to the VTA play a significant role in central reward processing [62,63]. We observed no effect on alcohol consumption following Ex-4 injection into either the ArcN or PVN; however, we observed a decrease in alcohol consumption following injection into the LH. Our data suggest that GLP-1 signaling mediates alcohol consumption primarily through changes in reward processing in areas such as the VTA, NAc, and even the LH. Conversely, although Ex-4 certainly affects mesocorticolimbic reward processing during food-seeking behavior, there is additional metabolic activity in the ArcN and PVN that has previously been discussed as one mechanism of action [6,20,21]. This is evidenced by the lack of effect on alcohol consumption and observed decline in operant behavior for sucrose pellets following injection into hypothalamic sites (ArcN and PVN) associated with metabolic function.

Our data suggest that of the hypothalamic sites tested, the LH is uniquely tied to reward processing. This aligns with optogenetic data that confirm the existence of a GABAergic pathway projecting from the LH to the VTA, which increases food- and reward-seeking when activated [64,65]. Further, López-Ferreras et al. [60] found that 55% of GLP-1R expressing LH neurons have projections to the VTA. Barbano et al. [62] found that food reward and appetitive motivation are differentially regulated by high- and low-frequency optogenetic stimulation of LH GABAergic neurons. They attempt to explain this by suggesting that differential co-release of neuropeptides at different stimulation rates may mediate this effect. Taken together, these data suggest a potential overlap between GLP-1 and GABAergic reward-modulating neuron systems. Evidence suggests that GLP-1 and GABAergic systems interact in the CA3 region of the hippocampus [66], although additional research is needed to explore this relationship within the LH.

In conclusion, our findings support a growing body of evidence that GLP-1 receptor agonists lower alcohol consumption in rats. Further, our data elucidate the specific areas where GLP-1 activity affects reward and metabolism. We identify novel regions of the CNS where GLP-1 activity modulates reward, including the DMHipp and the LH. This suggests that pharmacotherapeutic interventions may act on these brain regions to mediate drug reward. A currently prescribed GLP-1 agonist, liraglutide, attenuates alcohol intake, alcohol-induced locomotor stimulation, alcohol-induced accumbal dopamine release, alcohol-induced CPP, and withdrawal-induced anxiety in rodents [37,67]. Further research revealed that a weekly treatment (spanning 5 or 9 weeks) with a long-acting GLP-1 agonist, dulaglutide, reduced alcohol consumption and preference in both male and female rats [38]. Moreover, exenatide, a GLP-1 agonist, is currently being used in clinical trials to assess the long-term impact of GLP-1 agonists on alcohol intake in human adults, demonstrating the clinical relevance of this line of research [68].

## 4. Materials and Methods

### 4.1. Animals

Adult male Sprague Dawley rats (*N* = 64; *n* = 8 per anatomical site) obtained from Envigo Laboratories (Madison, WI, USA) were initially pair-housed in polypropylene cages and maintained on a 12 h light/dark cycle (lights out at 1300 h). Rats had free access to food (LabDiet, St. Louis, MO, USA) and water. After surgical guide implants were inserted, rats were individually housed. Colony temperature was maintained at 22 + 2 °C. Behavioral testing was conducted during the dark cycle. All experimental procedures were approved by the Institutional Animal Care and Use Committee of Reed College (IACUC, A4425-01; Protocol #RCPJC1820, Date of Approval 10 May 2018).

### 4.2. Drug

The GLP-1 analogue, exendin-4, was obtained from Tocris (Minneapolis, MN, USA). The compound was dissolved in sterile isotonic saline (0.15 M NaCl) immediately before treatment. For all microinjections, the volume of Ex-4 in solution was 0.2 µL. Infusions were administered over a 3 min period. Threshold and subthreshold doses were selected from pilot testing in our lab as well as previously published reports [42,43] illustrating motivational effects in rodents in the absence of disruptive behaviors. The specific doses of Ex-4 were as follows: 0 µg, 0.005 µg, 0.05 µg, and 0.5 µg.

### 4.3. Stereotaxic Surgery

Adult male rats weighing 270–310 g were anesthetized with a co-treatment of ketamine (100 mg/kg IP) and xylazine (5 mg/kg IP) and mounted into the Kopf stereotaxic apparatus with the incisor bar set 3.5 mm below the interaural line. All rats were unilaterally implanted with stainless steel guide cannulae (PlasticsOne, Roanoke, VA, USA). Cannulae were implanted dorsal to the target site as specified below [44]. This was done to minimize collateral damage to adjacent tissue. The following coordinates were used for a given structure: VTA posterior 6.8 mm, lateral +/− 0.6 mm, and ventral −4.6 mm. The VTA microinjector projected 4 mm beyond the base of the guide when it was inserted into the brain for Ex-4 delivery. For the NAcC, the coordinates were +1.7 mm, +/− 1.6 mm, and −4 mm, with the microinjector protruding 2 mm beyond the guide. In NAcS rats, the stereotaxic coordinates were +1.7 mm, +/− 0.8 mm, and −4.8 mm with a microinjector extension of 2 mm. BLA coordinates were −2.76 mm, +/− 4.7 mm, and 4.4 mm with a 4 mm microinjector projection. DMHipp coordinates were −2.28 mm, +/− 2 mm, and −2 mm with a 1.5 microinjector extension. Finally, in the hypothalamus, the ArcN coordinates were −2.3 mm, +/− 2 mm, and 5.7 mm with a 4 mm microinjector projection, whereas PVN coordinates were -1.8 mm, +/− 3 mm, and 4 mm with a 4 mm microinjector projection, and LH coordinates were −2.8 mm, +/− 1.5 mm, and −4.8 mm with a 4 mm microinjector projection used. Guide cannula assemblies were secured with acrylic cement and with three stainless steel anchor screws. Stylets were changed regularly to maintain patency. Rats were allowed to recover from surgery for at least two weeks before testing was begun.

### 4.4. Design and Procedure

#### 4.4.1. Alcohol Consummatory Investigations

Rats were habituated to a two-bottle intermittent choice paradigm [5,18,19,43], in which animals gained access to gradually increasing concentrations of alcohol every other day over the course of 12 weeks until consumption of 6% alcohol stabilized. During this time, food and water were freely available. All testing was conducted in the home cage for a 2 h period during the early portion of the dark cycle. With this method of gradual alcohol exposure, rats were not forced to consume high concentrations of alcohol that can be aversive. Instead, they acquired a gradual and stable consumption pattern. On the day of testing, rats were administered the appropriate dose of Ex-4, determined via a randomization protocol, and returned to their home cage with pre-weighed bottles of 6% alcohol by volume. Intakes were measured 2 h later.

#### 4.4.2. Operant Conditioning Paradigm

Rats were initially trained on a progressive ratio 3 (PR3) reinforcement paradigm with banana-flavored sucrose pellet reinforcers (Product Number F20024, Bio-Serve, Noyes, Lancaster, NH, USA). Specifically, when a rat responds by pressing the lever successfully, it receives a single sucrose pellet. In order to receive the next sucrose pellet, the rat must now respond by pressing the lever 4 times. Therefore, each rat must increase its response by an additional 3 lever presses in order to receive further reinforcement. This arrangement, therefore, is one in which a reinforcer is given after the completion of a specific number of responses and where the number of responses required from the rat increases after each reinforcement. Training sessions were conducted every day for a 2-week period in order to first establish consistent responding by the rat. Each rat was tested during a 2 h period during the early portion of the dark cycle. Methodological details outlining training and manual shaping have been described previously [43,69]. During actual training, animals were first exposed to a single fixed interval (FI) session with a sucrose pellet delivered every 30 s. Following one session of FI training, rats were then manually shaped in order to respond to a fixed ratio 1 (FR1) schedule. Here, each correct response was rewarded with a sucrose pellet. After responding consistently to the FR1, the schedule of reinforcement was gradually increased to fixed-ratio 10 (FR10) over three sessions. At this point, rats were placed on the PR3 schedule until each rat exhibited stabilized operant behavior. As in the alcohol study, a repeated measures design was used where rodents were subjected to each dosage condition administered in a randomized order. Subsequent test sessions were separated by at least four non-injection days. The test session for individual experiments lasted 30 min, and during this time, the number of reinforcers acquired was measured. Ex-4 was administered at doses ranging from 0–0.5 µg into the targeted anatomical structure.

#### 4.4.3. Histological and Statistical Analyses 

Histological sites were confirmed via histological examination. Immediately prior to brain extractions, injections of 0.2 µL of black ink were administered into target areas. Tissue was then fixed in 10% buffered formalin. Brains were cut coronally through the region of interest at 40 µm sections and stained with Cresyl violet. Sections were examined using light microscopy (Olympus Plan2x/0.05) and viewed relative to Paxino’s and Watson’s stereotaxic atlas [44]. Representative placements are shown in the results section in Figure 1. For statistical evaluations, data were analyzed using one-way repeated measures analyses of variance (ANOVA) with the criterion for statistical significance set at *p* < 0.05. Specific comparisons between means were carried out using post hoc Tukey tests where appropriate.
